# Supplementary material for: Phosphoregulated orthogonal signal transduction in mammalian cells
Source: Nat Commun. 2020 Jun 18;11:3085. doi: 10.1038/s41467-020-16895-1 (PMC7303213; doi:10.1038/s41467-020-16895-1)
Supplement: Supplementary file 1 — Supplementary Information [file 41467_2020_16895_MOESM1_ESM.docx]

**Supplementary Table 1.**

**Plasmid table**

|  |  |  |  |  |
| --- | --- | --- | --- | --- |
| **Plasmid** | **Description** | **Cloning strategy** | **Experiment** | **Reference /**  **GenBank: / sequence link^1^** |
| MKP37 | P_hCMV_-TetR-ELK1-pA  Mammalian expression vector for the MAPK reporter protein TetR-ELK1 | Keeley et al.^2^ | Supplementary Figure 4i  used in combination with a TetR reporter plasmid to monitor activity of the MAPK pathway and to exclude POST crosstalk. | Keeley et al.^2^ |
| pAB149 | P_hCMV_-DcuR-TetR-pA  Mammalian expression plasmid encoding DcuR fused to a C-terminal TetR domain. | Insert: DcuR from pMS9  Enzymes: EcoRI/XbaI  Backbone: pTS1106 (P_hCMV_-TetR-pA, unpublished)  Enzymes: EcoRI/SpeI | Figure 4c, 5c  Used in combination with 3xNLS-DcuR-VPR and a TetR reporter plasmid to monitor activity as DNA binding module for the two-hybrid OGR design, consisting of a TetR-DcuR and a DcuR-VPR fusion. | This work  GenBank: MT267299  <https://benchling.com/s/seq-9FLwtgUvRsplOFilyDey> |
| pAB150 | P_hCMV_-3xNLS-DcuR-VPR-pA  Mammalian expression plasmid encoding DcuR fused to a 3xNLS N-terminal domain and to a C-terminal VPR transactivator domain. | Insert: VPR from pVH15 (P_hCMV_-VPR-pA, unpublished)  Enzymes: SpeI/HindIII  Backbone: pAB148 (P_hCMV_--3xNLS-DcuR-pA, unpublished)  Enzymes: XbaI/HindIII. | Figure 4c, 5c  Used in combination with DcuR-TetR and a TetR reporter plasmid to monitor activity as a transactivator for the two-hybrid OGR design, consisting of a TetR-DcuR and a DcuR-VPR fusion. | This work  GenBank: MT267300  <https://benchling.com/s/seq-9lXubSMflJl8oarKHztp> |
| pCK53 | P_Cre_-SEAP-pA  Mammalian reporter plasmid containing Cre binding sites upstream of a minimal promoter driving SEAP expression. | Kemmer et al.^3^ | Supplementary Figure 4f  Used to monitor activity of the cAMP pathway and to exclude POST crosstalk. | Kemmer et al. ^3^ |
| pDF145 | P_T7_-SpAH-Env140ac  In vitro RNA production vector without mammalian promoter activity. | Ausländer et al.^4^ | Used in all experiments to reduce the amount of expression vectors, while retaining optimized DNA to PEI ratios for best transfection efficiency. | Ausländer et al.^4^ |
| pKR32 | P_NF-κB_-SEAP-pA  Mammalian reporter plasmid containing NF-κB binding sites upstream of a minimal promoter driving SEAP expression. | Schukur et al.^5^ | Supplementary Figure 4e  Used to monitor activity of the NF-κB pathway and to exclude POST crosstalk. | Schukur et al.^5^ |
| pLeo1216 | DcuR-RE_4x_-P_hCMVmin_-SEAP-pA  Mammalian reporter plasmid containing 4 DcuR binding sites (response elements; RE) upstream of a minimal hCMV promoter (P_hCMVmin_) driving SEAP expression. DcuR-RE_8x_-P_hCMVmin_-SEAP-pA | Insert: oligo annealing of oLeo818 (tcgacctgcagccagcactttttaaagttttgtaatcaggcgcggccggcgctatgcagcactttttaaagttttgtaatcactcgaga),  oLeo819 (cgcgtctcgagtgattacaaaactttaaaaagtgctgcatagcgccggccgcgcctgattacaaaactttaaaaagtgctggctgcagg)  Backbone: pMS8 (DcuR-RE_2x_-P_hCMVmin_-SEAP-pA)  Enzymes: MluI/XhoI | Used for cloning | This work  GenBank: MT267301  https://benchling.com/s/seq-j3VtHxAuDFBb65CQbkCC |
| pLeo1227 | DcuR-RE_6x_-P_hCMVmin_-SEAP-pA  Mammalian reporter plasmid containing 6 DcuR binding sites (response elements; RE) upstream of a minimal hCMV promoter (P_hCMVmin_) driving SEAP expression. | Insert: oligo annealing of oLeo818 (tcgacctgcagccagcactttttaaagttttgtaatcaggcgcggccggcgctatgcagcactttttaaagttttgtaatcactcgaga),  oLeo819 (cgcgtctcgagtgattacaaaactttaaaaagtgctgcatagcgccggccgcgcctgattacaaaactttaaaaagtgctggctgcagg)  Backbone: pLeo1216 (DcuR-RE_4x_-P_hCMVmin_-SEAP-pA)  Enzymes: MluI/XhoI | Used for cloning | This work  GenBank: MT267302  https://benchling.com/s/seq-SvOkrZvsbkuGOXr5XUxV |
| pLeo1228 | DcuR-RE_8x_-P_hCMVmin_-SEAP-pA  Mammalian reporter plasmid containing 8 DcuR binding sites (response elements; RE) upstream of a minimal hCMV promoter (P_hCMVmin_) driving SEAP expression. | Insert: DNA oligo annealing  DNA oligo: oLeo818 (tcgacctgcagccagcactttttaaagttttgtaatcaggcgcggccggcgctatgcagcactttttaaagttttgtaatcactcgaga),  oLeo819 (cgcgtctcgagtgattacaaaactttaaaaagtgctgcatagcgccggccgcgcctgattacaaaactttaaaaagtgctggctgcagg)  Backbone: pLeo1227 (DcuR-RE_6x_-P_hCMVmin_-SEAP-pA)  Enzymes: MluI/XhoI | Figures 2b, 3b 5 a,b, 6b, Supplementary Figures 1, 2 4a-d, 5  Used to quantify POST activity in mammalian cells with a secreted reporter readout. | This work  GenBank: MT267303  https://benchling.com/s/seq-UWPHDX3wqi3HlBLjiVzK |
| pLeo1231 | P_SV40_-DcuS_324-543_-pA  Mammalian expression plasmid encoding the DcuS intracellular domain DcuS_324-543_. | Insert: PCR amplification of DcuS_324-543_ from pMS79  Primers: oMS97 (ttcgaagcggaattcaccatgactagtaaaactgaagtacgtaaactgatgcagcg), oMS36 (ccggtggatccgctagcactagctctgttcgacctctccccgtc) Enzymes: SpeI/BamHI  Backbone: pMS79 (P_SV40_-DcuS_203-543_-pA)  Enzymes: SpeI/BamHI | Figure 2b  Testing basal activity of cytosolically expressed DcuS_324-543_. | This work  GenBank: MT267304  https://benchling.com/s/seq-H1fQlWvrga56yxUkHC40 |
| pLeo1242 | P_SV40_-DcuS_330-543_-pA  Mammalian expression plasmid encoding the DcuS intracellular domain DcuS_330-543_. | Insert: PCR amplification of DcuS_330-543_ from pMS79  Primers: oLeo824(aaccgaattcaccatgactagtctgatgcagcgactcgacg),  oMS36 (ccggtggatccgctagcactagctctgttcgacctctccccgtc)  Enzymes: SpeI/BamHI  Backbone: pMS79 (P_SV40_-DcuS_203-543_-pA)  Enzymes: SpeI/BamHI | Figure 2b  Testing basal activity of cytosolically expressed DcuS_330-543_. | This work  GenBank: MT267305  https://benchling.com/s/seq-gssYcYGRfiTD1JukanRD |
| pLeo1243 | P_SV40_-DcuS_334-543_-pA  Mammalian expression plasmid encoding the DcuS intracellular domain DcuS_334-543_. | Insert: PCR amplification of DcuS_334-543_ from pMS79  Primers: oMS80 (cgaagcggaattcaccatgactagttatgctgacgcacttcgtgaacg), oMS36 (ccggtggatccgctagcactagctctgttcgacctctccccgtc)  Enzymes: SpeI/BamHI  Backbone: pMS79 (P_SV40_-DcuS_203-543_-pA)  Enzymes: SpeI/BamHI | Figure 2b  Testing basal activity of cytosolically expressed DcuS_334-543_. | This work  GenBank: MT267306  https://benchling.com/s/seq-vLm6UInssyqsdvxg1NeD |
| pLeo1246 | P_SV40_-acV_H_H-GGGGS-DcuS_330-543_-pA  Mammalian expression plasmid encoding DcuS_330-543_ fused to an N-terminal nanobody that dimerizes in the presence of caffeine. | Insert: DcuS_330-543_ from pLeo1242  Enzymes: SpeI/HindIII  Backbone: pLeo1230 (P_SV40_-GGGGS-acV_H_H-pA, unpublished)  Enzymes: SpeI/HindIII | Figure 3b, 5a  Expression of an orthogonal receptor kinase (ORK) for testing POST in mammalian cells. | This work  GenBank: MT267307  https://benchling.com/s/seq-eBWl7K66hIa7egCbZNjR |
| pLeo1247 | P_SV40_-acV_H_H-GGGGS-DcuS_334-543_-pA  Mammalian expression plasmid encoding DcuS_334-543_ fused to an N-terminal nanobody that dimerizes in the presence of caffeine. | Insert: DcuS_334-543_ from pLeo1243  Enzymes: SpeI/HindIII  Backbone: pMSBB100 (P_SV40_-acV_H_H-GGGGS-pA, unpublished)  Enzymes: SpeI/HindIII | Figure 3b  Expression of an orthogonal receptor kinase (ORK) for testing POST in mammalian cells. | This work  GenBank: MT267308  https://benchling.com/s/seq-Z92EWUlHLzfWfItXMImG |
| pLeo1248 | P_SV40_-acV_H_H-GGGGS-DcuS_340-543_-pA  Mammalian expression plasmid encoding DcuS_340-543_ fused to an N-terminal nanobody that dimerizes in the presence of caffeine. | Insert: DcuS_340-543_ from pMS75  Enzymes: SpeI/HindIII  Backbone: pMSBB100 (P_SV40_-acV_H_H-GGGGS-pA, unpublished)  Enzymes: SpeI/HindIII | Figure 3b  Expression of an orthogonal receptor kinase (ORK) for testing POST in mammalian cells. | This work  GenBank: MT267309  https://benchling.com/s/seq-ppEJCKQbEI6ikiijICeW |
| pLeo1249 | P_hCMV_-NLS-acV_H_H-GGGGS-DcuS_324-543_-pA  Mammalian expression plasmid encoding NLS-DcuS_324-543_ fused to an N-terminal nanobody that dimerizes in the presence of caffeine. | Insert: VHH-DcuS_324-543_ from pMS247  Enzymes: SpeI/HindIII  Backbone: P_hcMV_-NLS (P_hCMV_-NLS-MCS-pA, unpublished)  Enzymes: NheI/HindIII | Figure 4c  Expression of an orthogonal receptor kinase (ORK) for activating the two hybrid OGR design. | This work  GenBank: MT267310  https://benchling.com/s/seq-WuQYqINGCzX0TJH6Ayyh |
| pLeo1254 | P_SV40_-DcuS_324-543_-GGGGS-acV_H_H-pA  Mammalian expression plasmid encoding DcuS_324-543_ fused to a C-terminal nanobody that dimerizes in the presence of caffeine. | Insert: DcuS_324-543_ from pLeo1231  Enzymes: EcoRI/NheI  Backbone: pLeo1230 (P_SV40_-GGGGS-acV_H_H-pA, unpublished)  Enzymes: EcoRI/SpeI | Figure 3b, 5b  Expression of an orthogonal receptor kinase (ORK) for testing POST in mammalian cells. | This work  GenBank: MT267311  https://benchling.com/s/seq-K1GG7HqSm2ItNCHQuAVe |
| pLeo1259 | P_SV40_-DcuS_330-543_-GGGGS-acV_H_H-pA  Mammalian expression plasmid encoding DcuS_330-543_ fused to a C-terminal nanobody that dimerizes in the presence of caffeine. | Insert: DcuS_330-543_ from pLeo1242  Enzymes: EcoRI/NheI  Backbone: pLeo1230 (P_SV40_-GGGGS-acV_H_H-pA, unpublished)  Enzymes: EcoRI/SpeI | Figure 3b, 5b  Expression of an orthogonal receptor kinase (ORK) for testing POST in mammalian cells. | This work  GenBank: MT267312  https://benchling.com/s/seq-ALQwiuMKKKhydvMmwkjH |
| pLeo1260 | P_SV40_-DcuS_334-543_-GGGGS-acV_H_H-pA  Mammalian expression plasmid encoding DcuS_334-543_ fused to a C-terminal nanobody that dimerizes in the presence of caffeine. | Insert: DcuS_334-543_ from pLeo1243  Enzymes: EcoRI/NheI  Backbone: pLeo1230 (P_SV40_-GGGGS-acV_H_H-pA, unpublished)  Enzymes: EcoRI/SpeI | Figure 3b  Expression of an orthogonal receptor kinase (ORK) for testing POST in mammalian cells. | This work  GenBank: MT267313  https://benchling.com/s/seq-Yv5c8tmJSGlHRiMsx33B |
| pLeo1261 | P_SV40_-DcuS_340-543_-GGGGS-acV_H_H-pA  Mammalian expression plasmid encoding DcuS_340-543_ fused to an C-terminal nanobody that dimerizes in the presence of caffeine. | Insert: DcuS_340-543_ from pMS75  Enzymes: EcoRI/NheI  Backbone: pLeo1230 (P_SV40_-GGGGS-acV_H_H-pA, unpublished)  Enzymes: EcoRI/SpeI | Figure 3b  Expression of an orthogonal receptor kinase (ORK) for testing POST in mammalian cells. | This work  GenBank: MT267314  https://benchling.com/s/seq-FufEafsa9u6IipKkgpHa |
| pLeo1286 | P_SV40_-acV_H_H-GGGGS-DcuS_324-543_-GGGGS-acV_H_HpA  Mammalian expression plasmid encoding DcuS_324-543_ fused to a C-terminal and N-terminal nanobody that dimerizes in the presence of caffeine. | Insert: V_H_H-DcuS_324-543_ from pMS247  Enzymes: EcoRI/NheI  Backbone: pLeo1230 (P_SV40_-GGGGS-acV_H_H-pA, unpublished)  Enzymes: EcoRI/SpeI | Figure 5c, Supplementary Figure 4d-i  Expression of an orthogonal receptor kinase (ORK) for testing POST in mammalian cells. | This work  GenBank: MT267315  https://benchling.com/s/seq-tBUYHp0onCNKFlH9tDb7 |
| pLeox1237 | P_SV40_-acV_H_H-GGGGS-DcuR-VP16-pA  Mammalian expression plasmid encoding a fusion protein of acV_H_H to DcuR. | Insert: acV_H_H from pMSBB100 (P_SV40_-acV_H_H-GGGGS-pA; unpublished)  Enzymes: EcoRI/NheI  Backbone: pMS66  Enzymes: EcoRI/SpeI | Supplementary Figure 2  Expression of an acV_H_H-DcuR fusion without the requirement of an ORK for activation, to confirm a dimerization dependent activation mechanism of DcuR. | This work  GenBank: MT267316  https://benchling.com/s/seq-QMJCwtvocArs3LrZeI3M |
| pMF111 | P_tetO7_-SEAP-pA  Mammalian TetR reporter plasmid to monitor TetR-Elk1 induced SEAP expression | Fussenegger et al. ^6^ | Supplementary Figure 4i  Used in combination with MKP37 to monitor MAPK activity and to exclude POST crosstalk. | Fussenegger et al. ^6^ |
| pMS8 | (DcuR-RE2_x_-P_hCMVmin_-SEAP-pA)  Mammalian reporter plasmid containing 2 DcuR binding sites (response elements; RE) upstream of a minimal hCMV promoter (P_hCMVmin_) driving SEAP expression. DcuR-RE_8x_-P_hCMVmin_-SEAP-pA | Insert: oligo annealing of oLeo818 (tcgacctgcagccagcactttttaaagttttgtaatcaggcgcggccggcgctatgcagcactttttaaagttttgtaatcactcgaga),  oLeo819 (cgcgtctcgagtgattacaaaactttaaaaagtgctgcatagcgccggccgcgcctgattacaaaactttaaaaagtgctggctgcagg)  Backbone: pTS1015 (P_hCMVmin_-SEAP-pA, unpublished)  Enzymes: MluI/XhoI. | Used for cloning. | This work |
| pMS9 | P_hCMV_-DcuR-pA  Mammalian expression plasmid encoding DcuR. | Insert: DcuR amplified from XL10 gold bacteria  Primers: oMS12 (accatgactagtggtggttctggtatgatcaatgtattaattatcgatgacgacgcaatgg), oMS13 (ctcaaagcttctatctagaaccggtggatccgctagcttggcaatattgtttcagtagtgagtagtgttctgc)  Enzymes: SpeI/HindIII  Backbone: pLeo1086 (P_hCMV_-GFP-pA, unpublished)  Enzymes: SpeI/HindIII | Used for cloning. | This work |
| pMS13 | P_hCMV_-DcuR-VP16-pA  Mammalian expression plasmid encoding DcuR fused to a C-terminal VP16 transactivator domain. | Insert: DcuR from PMS9  Enzymes: EcoRI/XbaI  Backbone: BB3-VP16 (P_hCMV_-VP16-pA, unpublished)  Enzymes: EcoRI/SpeI | Used for cloning. | This work |
| pMS66 | P_SV40_-DcuR-VP16-pA  Mammalian expression plasmid encoding DcuR fused to a C-terminal VP16 transactivator domain. | Insert: DcuR-VP16 from PMS13  Enzymes: EcoRI/HindIII  Backbone: pLeo1087 (P_SV40_-GFP-pA, unpublished)  Enzymes: EcoRI/HindIII | Figures 2b, 3b 5 a,b, 6b, Supplementary Figures 1, 2 4a-i, 5  Expression of the response regulator for testing POST in mammalian cells. | This work  GenBank: MT267317  https://benchling.com/s/seq-9qKNEX7AWwgrO0nNJ8mQ |
| pMS75 | P_SV40_-DcuS_340-543_-pA  Mammalian expression plasmid encoding the DcuS intracellular domain DcuS_340-543_. | Insert: PCR amplification of DcuS_340-543_ from pMS79  Primers: oMS80 (cgaagcggaattcaccatgactagttatgctgacgcacttcgtgaacg), oMS36 (ccggtggatccgctagcactagctctgttcgacctctccccgtc)  Enzymes: SpeI/BamHI  Backbone: pMS79 (P_SV40_-DcuS_203-543_-pA)  Enzymes: SpeI/BamHI | Figure 2b  Testing basal activity of cytosolically expressed DcuS_340-543_. | This work  GenBank: MT267318  https://benchling.com/s/seq-L5dcJFgvY0CCaQeF7k5a |
| pMS79 | P_SV40_-DcuS_203-543_-pA  Mammalian expression plasmid encoding the DcuS intracellular domain DcuS_203-543_. | Insert: DcuS_203-543_ amplified from XL10 gold bacteria  Primers: oMS83 (agcggaattcaccatgactagtaaggtactgaaaaaaatccttttcggcctg), oMS36 (ccggtggatccgctagcactagctctgttcgacctctccccgtc)  Enzymes: SpeI/BamHI  Backbone: pLeo1087 (P_SV40_-GFP-pA, unpublished)  Enzymes: SpeI/BamHI | Figure 2b  Testing basal activity of cytosolically expressed DcuS_203-543_. | This work  GenBank: MT267319  https://benchling.com/s/seq-HIm3frE2eDSiWgWSHWrU |
| pMS247 | P_SV40_-acV_H_H-GGGGS-DcuS_324-543_-pA  Mammalian expression plasmid encoding DcuS_324-543_ fused to an N-terminal nanobody that dimerizes in the presence of caffeine. | Insert: DcuS_324-543_ from pLeo1231  Enzymes: SpeI/HindIII  Backbone: pMSBB100 (P_SV40_-acV_H_H-GGGGS-pA, unpublished)  Enzymes: SpeI/HindIII | Figure 2b, 5a, Supplementary Figure 4a-c  Expression of an orthogonal receptor kinase (ORK) for testing POST in mammalian cells. | This work  GenBank: MT267320  https://benchling.com/s/seq-ie9T9WvmesNMb3l9KPa7 |
| pMSx4 | OmpR-RE_2x_-P_hCMVmin_-SEAP-pA  Mammalian reporter plasmid containing 2 OmpR binding sites (response elements; RE) upstream of a minimal hCMV promoter (P_hCMVmin_) driving SEAP expression. | Primers: oMS3 (aacacctcgagatttacattttgaaacatctatagcgccggcatttacattttgaaacatctagctgcaggtcgagggtaggc), oLeo223(aaccaaaccggttagaaggcacagtcgagg)  Template: pLeo1228  Enzymes: XhoI/HindIII  Backbone: pLeo1228  Enzymes: XhoI/HindIII | Figure 7 b  Reporter plasmid for testing EnvZ/OmpR POST in mammalian cells. | This work  GenBank: MT267321  https://benchling.com/s/seq-XBIjNWYIjrTgzQzVVBB3 |
| pMSx5 | NarL-RE_2x_-P_hCMVmin_-SEAP-pA  Mammalian reporter plasmid containing 2 NarL binding sites (response elements; RE) upstream of a minimal hCMV promoter (P_hCMVmin_) driving SEAP expression. | Primers: oMS6 (cacaacctcgagtacccctataggggtatagcgccggctacccctataggggtagctgcaggtcgagggtaggc), oLeo223(aaccaaaccggttagaaggcacagtcgagg)  Template: pLeo1228  Enzymes: XhoI/HindIII  Backbone: pLeo1228  Enzymes: XhoI/HindIII | Figure 7 d  Reporter plasmid for testing NarX/NarL POST in mammalian cells. | This work  GenBank: MT267322  https://benchling.com/s/seq-4Fmt3SomCfPdU9l8BBd6 |
| pMSx11 | P_hCMV__OmpR-VP16-pA  Mammalian expression plasmid encoding OmpR-VP16 | Primers: oMS4(aagcggaattcaccatgactagtggtggttctggtatgcaagagaactacaagattctggtggtc), oMS5(aaaatggatccgctagctttagagccgtccggtacaaagacg)  Template: E. coli (XL10-Gold)  Enzymes: EcoRI/NheI  Backbone: BB3-VP16 (P_hCMV_-VP16-pA, unpublished)  Enzymes: EcoRI/SpeI | Used for cloning | This work |
| pMSx12 | P_hCMV__NarL-VP16-pA  Mammalian expression plasmid encoding NarL. | Primers: oMS10 (accggtggatccgctagcgaaaatgcgctcctgatgcacc), oMS11(agcggaattcaccatgactagtggtggttctggtatgagtaatcaggaaccggctactatcc)  Template: E. coli (XL10-Gold)  Enzymes: EcoRI/NheI  Backbone: BB3-VP16 (P_hCMV_-VP16-pA, unpublished)  Enzymes: EcoRI/SpeI | Used for cloning | This work |
| pMSx64 | P_sv40__OmpR-VP16-pA  Mammalian expression plasmid encoding OmpR-VP16 | Insert: OmpR-VP16 from pMS11  Enzymes: SpeI/HindIII  Backbone: pMSBB100 (P_SV40_-acV_H_H-GGGGS-pA, unpublished)  Enzymes: NheI/HindIII | Figure 7 b  Expression of the OmpR OGR for testing EnvZ/OmpR POST in mammalian cells. | This work  GenBank: MT267323  https://benchling.com/s/seq-fL77my1WOMyPdGJvkrkH |
| pMSx65 | P_sv40__NarL-VP16-pA  Mammalian expression plasmid encoding NarL fused to the VP16 transactivator domain. | Insert: NarL-VP16 from PMS12  Enzymes: SpeI/HindIII  Backbone: pMSBB100 (P_SV40_-acV_H_H-GGGGS-pA, unpublished) Enzymes: SpeI/HindIII | Figure 7 d  Expression of the NarL OGR for testing NarX/NarL POST in mammalian cells. | This work  GenBank: MT267324  https://benchling.com/s/seq-sWbgba0SvfKDDnZnDdo6 |
| pMSx78 | P_sv40__NarX_263-598_-pA  Mammalian expression plasmid encoding NarX_233-598._ | Primers: oMS79 (cgaagcggaattcaccatgactagtccgctgtgtgaacgcctgtc), oMS33(ccggtggatccgctagcactagcctcatgggtatctccttggacgtctg)  Template: pMS88  Enzymes: SpeI/HindIII  Backbone: pMS247 Enzymes: SpeI/HindIII | Figure 7 d  Truncation mutant without acV_H_H, used to test basal NarX_263-598_ activity and used as negative control for caffeine induced POST. | This work  GenBank: MT267325  https://benchling.com/s/seq-1oSGSUvwQ6RklAh6zKdA |
| pMSx80 | P_sv40__EnvZ_232-450_-pA  Mammalian expression plasmid encoding EnvZ_232-450_ | Primers: oMS74(ttcgaagcggaattcaccatgactagtgatgaccgcacgctgctgatg), oMS28(ccggtggatccgctagcactagccccttcttttgtcgtgccctgc)  Template: *E.coli* (BL-21)  Enzymes: SpeI/HindIII  Backbone: pMS247  Enzymes: SpeI/HindIII | Supplementary Figure 6  Truncation mutant without acV_H_H, used to test basal EnvZ_232-450_ activity. | This work  GenBank: MT267326  https://benchling.com/s/seq-dY3Hpi6yR9LqPw2zozEB |
| pMSx88 | P_sv40__NarX_233-598_-pA  Mammalian expression plasmid encoding NarX_263-598._ | Primers: oMS92 (ttcgaagcggaattcaccatgactagtgtacttgagcagcgggttcagg), oMS33(ccggtggatccgctagcactagcctcatgggtatctccttggacgtctg)  Template: *E.coli* (XL10-gold)  Enzymes: SpeI/HindIII  Backbone: pMS247 Enzymes: SpeI/HindIII | Figure 7 d  Truncation mutant without acV_H_H, used to test basal NarX_233-598_ activity and used as negative control for caffeine induced POST. | This work  GenBank: MT267327  https://benchling.com/s/seq-gYe8dXKWk0gQHAfPcMfk |
| pMSx119 | P_sv40__EnvZ_232-450;GQEMP:AS_-pA  Mammalian expression plasmid encoding EnvZ_232-450; GQEMP:AS_ | Primers: PCR1: oMS74, oMS69 (taatcaggatccgctagcggtgcgcaggtagtcgataaactg); PCR2: oMS28, oMS70(attaaactagtatggaaatggcggatcttaatgcagtactc)  Template: pMS80  Enzymes: PCR1: EcoRI/NheI; PCR2: SpeI/HindIII  Backbone: pMS80  Enzymes: EcoRI/HindIII | Figure 7 b  Truncation mutant without acV_H_H, used to test basal EnvZ_232-450;GQEMP:AS_ activity and used as negative control for caffeine induced POST. | This work  GenBank: MT267328  https://benchling.com/s/seq-wIQtCs8jQ1hNhCrer0aE |
| pMSx132 | P_sv40__acV_H_H-EnvZ_232-450;GQEMP:AS_-pA  Mammalian expression plasmid encoding acV_H_H-EnvZ_232-450; GQEMP:AS_ | Insert: EnvZ_232-450; GQEMP:AS_ from pMS119  Enzymes: SpeI/HindIII  Backbone: pMSBB100 (P_SV40_-acV_H_H-GGGGS-pA, unpublished)  Enzymes: NheI/HindIII | Figure 7 b  Expression of the EnvZ ORK for testing EnvZ/OmpR POST in mammalian cells. | This work  GenBank: MT267329  https://benchling.com/s/seq-0AzqtgfblyD0XNxGOnVU |
| pMSx228 | P_SV40_-FRB-GGGGS-DcuS_324-543_-pA  Mammalian expression plasmid encoding the FRB ORK | Insert: FRB-GGGGS from pMSBB95 (P_SV40_-FRB-GGGGS-pA; unpublished)  Enzymes: EcoRI/NheI  Backbone: pLeo1231  Enzymes: EcoRI/SpeI | Supplementary Figure 1  Expression of the FRB ORK for testing FRB/FKBP POST in mammalian cells. | This work  GenBank: MT267330  https://benchling.com/s/seq-nZa0YoqCM7HnSJifPhjS |
| pMSx230 | P_SV40_-FKBP-GGGGS-DcuS_324-543_-pA  Mammalian expression plasmid encoding the FKBP ORK | Insert: FKBP-GGGGS from pMSBB96 (P_SV40_-FRB-GGGGS-pA; unpublished)  Enzymes: EcoRI/NheI  Backbone: pLeo1231  Enzymes: EcoRI/SpeI | Supplementary Figure 1  Expression of the FKBP ORK for testing FRB/FKBP POST in mammalian cells. | This work  GenBank: MT267331  https://benchling.com/s/seq-4s107BpHoFvwecTS3Ti0 |
| pMSx245 | P_sv40__acV_H_H- NarX_263-598_-pA  Mammalian expression plasmid encoding NarX_233-598_ fused to an N-terminal nanobody that dimerizes in the presence of caffeine. | Insert: NarX_263-598_ from pMS78  Enzymes: SpeI/HindIII  Backbone: pMSBB100 (P_SV40_-acV_H_H-GGGGS-pA, unpublished)  Enzymes: SpeI/HindIII | Figure 7 d  Expression of the NarX ORK for testing NarX/NarL POST in mammalian cells. | This work  GenBank: MT267332  https://benchling.com/s/seq-2rTV9fWxxqce2a8LVlU2 |
| pMX57 | P_NFAT_-SEAP-pA  Mammalian reporter plasmid containing NFAT binding sites upstream of a minimal promoter driving SEAP expression. | Xie et al. ^7^ | Supplementary Figure 1i  Used to monitor NFAT activity and to exclude POST crosstalk. | Xie et al. ^7^ |
| pTS566 | P_STAT3_-SEAP-pA  Mammalian reporter plasmid containing STAT3 binding sites upstream of a minimal promoter driving SEAP expression. | Insert: oligo annealing of OTS739 (cgcgtctcgagatttcccgtaaatcgtcgagtggacatttcccgtaaatcgtcgagtggacatttcccgtaaatcgtcgagtggacatttcccgtaaatcgtcgagctgcagg),  OTS740 (tcgacctgcaggtcgacgatttacgggaaatgtccactcgacgatttacgggaaatgtccactcgacgatttacgggaaatgtccactcgacgatttacgggaaatctcgaga)  Backbone: pTS1015 (P_hCMVmin_-SEAP-pA, unpublished)  Enzymes: MluI/XhoI. | Supplementary Figure 1h  Used to monitor STAT3 activity and to exclude POST crosstalk. | This work  GenBank: MT267333  https://benchling.com/s/seq-bz1iHgarnBSj30pokrEo |
| pTS1017 | P_TetO7_-SEAP-pA  Mammalian reporter plasmid containing TetR binding sites upstream of a minimal promoter driving SEAP expression. | Insert: TETO_7_ (Auslander et al. ^8^)  primers: oTS733 (ctgaacgcgtccgtacacgcctaaagcatatacgttc)  oTS734: (cctcgacatactcgagtttactccctatc)  Backbone: pTS1015 (P_hCMVmin_-SEAP-pA, unpublished)  Enzymes: MluI/XhoI. | Supplementary Figure 1i  Reporter plasmid for the two-hybrid OGR design, consisting of a TETR-DcuR and a DcuR-VPR fusion. | This work  GenBank: MT267334  https://benchling.com/s/seq-0PesBuXSye3gNNFvsfJL |

| **Abbreviations** |
| --- |
| **acV_H_H**, anti-caffeine heavy chain antibody |
| **cAMP**, cyclic adenosine monophosphate |
| **CRE**, cAMP response element |
| **MAPK**, mitogen-activated protein kinase |
| **MCS**, multiple cloning site |
| **NF-κB**, nuclear factor kappa-light-chain-enhancer of activated B cells |
| **NFAT**, nuclear factor of activated T-cells |
| **NLS**, nuclear localization sequence |
| **OGR**, orthogonal gene expression regulator |
| **ORK**, orthogonal receptor kinase |
| **pA**, polyadenylation signal |
| **PEI**, polyethylenimine |
| **P_hCMV_**, human cytomegalovirus immediate early promoter |
| **P_hCMVmin_**, minimal human cytomegalovirus immediate early promoter |
| **POST**, phosphoregulated orthogonal signal transduction |
| **P_SV40_**, simian virus 40 derived promoter |
| **RE**, response elements |
| **SEAP**, human placental secreted alkaline phosphatase |
| **STAT3**, signal transducer and activator of transcription 3 |
| **TCS**, two-component system |
| **TetR**, Tet Repressor protein |
| **VPR**, VP64-p65-Rta (a fusion of 3 transactivator domains) |

**References**

1 Benchling [Biology Software].  Retrieved from  <https://benchling.com> (2020).

2 Keeley, M. B., Busch, J., Singh, R. & Abel, T. TetR hybrid transcription factors report cell signaling and are inhibited by doxycycline. *BioTechniques* **39**, 529-536 (2005).

3 Kemmer, C. *et al.* A designer network coordinating bovine artificial insemination by ovulation-triggered release of implanted sperms. *Journal of controlled release : official journal of the Controlled Release Society* **150**, 23-29, doi:10.1016/j.jconrel.2010.11.016 (2011).

4 Ausländer, S., Fuchs, D., Hürlemann, S., Ausländer, D. & Fussenegger, M. Engineering a ribozyme cleavage-induced split fluorescent aptamer complementation assay. *Nucleic Acids Res* **44**, e94, doi:10.1093/nar/gkw117 (2016).

5 Schukur, L., Geering, B., Charpin-El Hamri, G. & Fussenegger, M. Implantable synthetic cytokine converter cells with AND-gate logic treat experimental psoriasis. *Science translational medicine* **7**, 318ra201, doi:10.1126/scitranslmed.aac4964 (2015).

6 Fussenegger, M. *et al.* Streptogramin-based gene regulation systems for mammalian cells. *Nature biotechnology* **18**, 1203-1208, doi:10.1038/81208 (2000).

7 Xie, M. *et al.* beta-cell-mimetic designer cells provide closed-loop glycemic control. *Science* **354**, 1296-1301, doi:10.1126/science.aaf4006 (2016).

8 Auslander, D. *et al.* Programmable full-adder computations in communicating three-dimensional cell cultures. *Nature methods* **15**, 57-60, doi:10.1038/nmeth.4505 (2018).

Supplementary Table 2.

Statistics and parameters for Figure 5 and 6b

| The results tables from GraphPad Prism for representative figures and independent repeats, containing all parameters for the dose response curves, ordered by figures. | | | | | | | | | | |
| --- | --- | --- | --- | --- | --- | --- | --- | --- | --- | --- |
| **Figure 5 a** | | | | | | | | | | |
|  | AcV_H_H-DcuS_324-543_ | | AcV_H_H-DcuS_324-543_ | | AcV_H_H-DcuS_324-543_ | | AcV_H_H-DcuS_330-543_ | | AcV_H_H-DcuS_330-543_ | AcV_H_H-DcuS_330-543_ |
| **Sigmoidal, 4PL, X is concentration** |  | |  | |  | |  | |  |  |
| **Best-fit values** |  | |  | |  | |  | |  |  |
| Bottom | 48.14 | | 50.64 | | 39.56 | | 43.9 | | 48.21 | 26.49 |
| Top | 292.4 | | 319.5 | | 249.6 | | 292.1 | | 233.1 | 219.3 |
| IC50 | 0.4662 | | 1.508 | | 1.096 | | 0.6977 | | 0.4017 | 1.83 |
| HillSlope | 0.7058 | | 0.5118 | | 0.4854 | | 0.4906 | | 0.805 | 0.3759 |
| logIC50 | -0.3314 | | 0.1785 | | 0.03969 | | -0.1564 | | -0.3961 | 0.2624 |
| Span | 244.3 | | 268.8 | | 210.1 | | 248.2 | | 184.9 | 192.8 |
| **95% CI (profile likelihood)** |  | |  | |  | |  | |  |  |
| Bottom | 29.66 to 65.24 | | 28.41 to 70.21 | | 30.59 to 47.89 | | 6.588 to 72.78 | | 35.76 to 59.99 | 3.678 to 43.96 |
| Top | 266.1 to 328.3 | | 278.4 to 407.2 | | 231.7 to 275.5 | | 241.3 to 457.5 | | 216.7 to 252.7 | 176.5 to 503.8 |
| IC50 | 0.2057 to 1.234 | | 0.4477 to 11.23 | | 0.5612 to 2.527 | | 0.1132 to 42.33 | | 0.2026 to 0.8114 | 0.2529 to 6538 |
| HillSlope | 0.4249 to 1.288 | | 0.2930 to 0.9106 | | 0.3661 to 0.6433 | | 0.2071 to 1.470 | | 0.5109 to 1.291 | 0.1684 to 0.7972 |
| logIC50 | -0.6868 to 0.09138 | | -0.3490 to 1.050 | | -0.2509 to 0.4026 | | -0.9462 to 1.627 | | -0.6934 to -0.09078 | -0.5970 to 3.815 |
| **Goodness of Fit** |  | |  | |  | |  | |  |  |
| Degrees of Freedom | 26 | | 26 | | 26 | | 26 | | 26 | 26 |
| R squared | 0.933 | | 0.915 | | 0.9763 | | 0.826 | | 0.9454 | 0.8776 |
| Sum of Squares | 22501 | | 28012 | | 4474 | | 56548 | | 10897 | 17509 |
| Sy.x | 29.42 | | 32.82 | | 13.12 | | 46.64 | | 20.47 | 25.95 |
|  |  | |  | |  | |  | |  |  |
| **Number of points** |  | |  | |  | |  | |  |  |
| # of X values | 30 | | 30 | | 30 | | 30 | | 30 | 30 |
| # Y values analyzed | 30 | | 30 | | 30 | | 30 | | 30 | 30 |
| **Figure 5 b** | | | | | | | | | | |
|  | DcuS_324-543_-AcV_H_H | | DcuS_324-543_-AcV_H_H | | DcuS_324-543_-AcV_H_H | | DcuS_330-543_-AcV_H_H | | DcuS_330-543_-AcV_H_H | DcuS_330-543_-AcV_H_H |
| **Sigmoidal, 4PL, X is concentration** |  | |  | |  | |  | |  |  |
| **Best-fit values** |  | |  | |  | |  | |  |  |
| Bottom | 44.15 | | 36.54 | | 55.32 | | 21.43 | | 21.67 | 23.4 |
| Top | 217 | | 184.5 | | 288.8 | | 112.2 | | 133.4 | 174.2 |
| IC50 | 0.5723 | | 0.3387 | | 0.1501 | | 2.56 | | 0.6598 | 3.681 |
| HillSlope | 0.5776 | | 0.5421 | | 0.669 | | 0.4206 | | 0.5864 | 0.4146 |
| logIC50 | -0.2423 | | -0.4702 | | -0.8236 | | 0.4082 | | -0.1806 | 0.566 |
| Span | 172.8 | | 147.9 | | 233.5 | | 90.78 | | 111.7 | 150.8 |
| **95% CI (profile likelihood)** |  | |  | |  | |  | |  |  |
| Bottom | 22.65 to 61.52 | | 19.80 to 50.11 | | 41.94 to 67.80 | | 13.24 to 28.01 | | 11.74 to 30.46 | 10.61 to 33.72 |
| Top | 188.3 to 296.4 | | 164.4 to 229.9 | | 273.6 to 305.6 | | 94.79 to 175.8 | | 118.7 to 160.6 | 145.1 to 290.0 |
| IC50 | 0.1475 to 9.347 | | 0.1019 to 2.403 | | 0.08292 to 0.2696 | | 0.5364 to 167.1 | | 0.2271 to 3.059 | 0.7959 to 310.1 |
| HillSlope | 0.2507 to 1.398 | | 0.2701 to 1.061 | | 0.4936 to 0.9303 | | 0.2179 to 0.7656 | | 0.3252 to 1.088 | 0.2171 to 0.7384 |
| logIC50 | -0.8313 to 0.9707 | | -0.9919 to 0.3807 | | -1.081 to -0.5693 | | -0.2705 to 2.223 | | -0.6438 to 0.4855 | -0.09912 to 2.492 |
| **Goodness of Fit** |  | |  | |  | |  | |  |  |
| Degrees of Freedom | 26 | | 26 | | 26 | | 26 | | 26 | 26 |
| R squared | 0.8684 | | 0.9024 | | 0.9677 | | 0.9104 | | 0.9143 | 0.9161 |
| Sum of Squares | 21677 | | 11365 | | 9852 | | 2849 | | 5582 | 6936 |
| Sy.x | 28.87 | | 20.91 | | 19.47 | | 10.47 | | 14.65 | 16.33 |
|  |  | |  | |  | |  | |  |  |
| **Number of points** |  | |  | |  | |  | |  |  |
| # of X values | 30 | | 30 | | 30 | | 30 | | 30 | 30 |
| # Y values analyzed | 30 | | 30 | | 30 | | 30 | | 30 | 30 |
| **Figure 5 c** | | | | | | | | | | |
|  | | Two-hybrid OGR | | Two-hybrid OGR | | Two-hybrid OGR | |  |  |  |
| **Sigmoidal, 4PL, X is concentration** | |  | |  | |  | |  |  |  |
| **Best-fit values** | |  | |  | |  | |  |  |  |
| Bottom | | 21.82 | | 15.51 | | 24.18 | |  |  |  |
| Top | | 139.3 | | 120.2 | | 139.1 | |  |  |  |
| IC50 | | 0.09563 | | 0.2271 | | 0.1174 | |  |  |  |
| HillSlope | | 0.9102 | | 0.8769 | | 0.7918 | |  |  |  |
| logIC50 | | -1.019 | | -0.6438 | | -0.9302 | |  |  |  |
| Span | | 117.5 | | 104.7 | | 115 | |  |  |  |
| **95% CI (profile likelihood)** | |  | |  | |  | |  |  |  |
| Bottom | | 18.41 to 25.16 | | 12.07 to 18.85 | | 19.38 to 28.78 | |  |  |  |
| Top | | 135.1 to 143.6 | | 115.5 to 125.1 | | 133.2 to 145.7 | |  |  |  |
| IC50 | | 0.07924 to 0.1157 | | 0.1802 to 0.2865 | | 0.08785 to 0.1581 | |  |  |  |
| HillSlope | | 0.7873 to 1.057 | | 0.7425 to 1.040 | | 0.6441 to 0.9780 | |  |  |  |
| logIC50 | | -1.101 to -0.9366 | | -0.7442 to -0.5429 | | -1.056 to -0.8009 | |  |  |  |
| **Goodness of Fit** | |  | |  | |  | |  |  |  |
| Degrees of Freedom | | 26 | | 26 | | 26 | |  |  |  |
| R squared | | 0.9912 | | 0.9871 | | 0.9826 | |  |  |  |
| Sum of Squares | | 591.7 | | 644.3 | | 1057 | |  |  |  |
| Sy.x | | 4.77 | | 4.978 | | 6.375 | |  |  |  |
|  | |  | |  | |  | |  |  |  |
| **Number of points** | |  | |  | |  | |  |  |  |
| # of X values | | 30 | | 30 | | 30 | |  |  |  |
| # Y values analyzed | | 30 | | 30 | | 30 | |  |  |  |
| **Figure 6b** | | | | | | | |  |  |  |
|  | | dual ORK | | dual ORK | | dual ORK | |  |  |  |
| **Sigmoidal, 4PL, X is concentration** | |  | |  | |  | |  |  |  |
| **Best-fit values** | |  | |  | |  | |  |  |  |
| Bottom | | 29.15 | | 31.15 | | 49.65 | |  |  |  |
| Top | | 238 | | 341.3 | | 315.3 | |  |  |  |
| IC50 | | 0.3856 | | 0.4531 | | 0.6577 | |  |  |  |
| HillSlope | | 1.076 | | 0.8727 | | 1.465 | |  |  |  |
| logIC50 | | -0.4138 | | -0.3438 | | -0.182 | |  |  |  |
| Span | | 208.8 | | 310.2 | | 265.7 | |  |  |  |
| **95% CI (profile likelihood)** | |  | |  | |  | |  |  |  |
| Bottom | | 12.61 to 44.84 | | -8.246 to 65.52 | | 38.55 to 60.61 | |  |  |  |
| Top | | 219.9 to 258.9 | | 295.3 to 408.9 | | 300.5 to 331.0 | |  |  |  |
| IC50 | | 0.2506 to 0.6360 | | 0.2156 to 1.337 | | 0.4643 to ??? | |  |  |  |
| HillSlope | | 0.6784 to 1.953 | | 0.4193 to 3.269 | | 0.9522 to ??? | |  |  |  |
| logIC50 | | -0.6010 to -0.1965 | | -0.6664 to 0.1260 | | -0.3332 to ??? | |  |  |  |
| **Goodness of Fit** | |  | |  | |  | |  |  |  |
| Degrees of Freedom | | 26 | | 26 | | 26 | |  |  |  |
| R squared | | 0.945 | | 0.8831 | | 0.9749 | |  |  |  |
| Sum of Squares | | 13408 | | 62193 | | 11053 | |  |  |  |
| Sy.x | | 22.71 | | 48.91 | | 20.62 | |  |  |  |
|  | |  | |  | |  | |  |  |  |
| **Number of points** | |  | |  | |  | |  |  |  |
| # of X values | | 30 | | 30 | | 60 | |  |  |  |
| # Y values analyzed | | 30 | | 30 | | 30 | |  |  |  |

Supplementary Table 3

Statistics for Supplementary Figure 3. "Statistical significance determined using the Holm-Sidak method, with alpha = 0.05. Each row was analyzed individually, without assuming a consistent SD. Number of t-tests: 5."*

*This table and explanation were copied from the results section of GraphPad Prism 8 and a column for the labels (hours) and thresholds for the * used in Supplementary Figure 3 were added. The Holm-Sidak method consists of multiple two-sided t-tests and corrects for multiple comparisons.

| hours | Significant? | P value | Mean of no inducer | Mean of caffeine 100 µM | Difference | SE of difference | t ratio | df | Adjusted P Value | Significance | |
| --- | --- | --- | --- | --- | --- | --- | --- | --- | --- | --- | --- |
| 2 | No | 0.370856 | 0.4895 | 0.5987 | -0.1092 | 0.1084 | 1.007 | 4 | 0.370856 | P > 0.05 | Ns |
| 4 | Yes | 0.002393 | 0.7619 | 3.912 | -3.15 | 0.4608 | 6.838 | 4 | 0.004781 | P ≤ 0.01 | ** |
| 6 | Yes | 0.000138 | 1.439 | 11.18 | -9.738 | 0.6796 | 14.33 | 4 | 0.000689 | P ≤ 0.001 | *** |
| 8 | Yes | 0.001166 | 2.176 | 19.04 | -16.87 | 2.039 | 8.272 | 4 | 0.003493 | P ≤ 0.01 | ** |
| 10 | Yes | 0.000187 | 2.704 | 37.55 | -34.85 | 2.628 | 13.26 | 4 | 0.000748 | P ≤ 0.001 | *** |

Supplementary Table 4.

Transfection table

Figure 2b

| **label** | **PEI per 6 wells (µg)** | **Plasmid amount (DNA per 6 wells in ng)** | | | |
| --- | --- | --- | --- | --- | --- |
|  |  | **Receptor** | **Response regulator** | **Reporter** | **Fill up** |
| DcuS_203-543_ | 3.75 | pMS79: P_SV40_-DcuS_203-543_-pA | pMS66: P_SV40_-DcuR-VP16-pA | pLeo1228: DcuR-RE_8x_-P_hCMVmin_-SEAP-pA | pDF145 |
|  |  | 50 | 200 | 150 | 350 |
| DcuS_324-543_ | 3.75 | pLeo1231: P_SV40_-DcuS_324-543_-pA | pMS66: P_SV40_-DcuR-VP16-pA | pLeo1228: DcuR-RE_8x_-P_hCMVmin_-SEAP-pA | pDF145 |
|  |  | 50 | 200 | 150 | 350 |
| DcuS_330-543_ | 3.75 | pLeo1242: P_SV40_-DcuS_330-543_-pA | pMS66: P_SV40_-DcuR-VP16-pA | pLeo1228: DcuR-RE_8x_-P_hCMVmin_-SEAP-pA | pDF145 |
|  |  | 50 | 200 | 150 | 350 |
| DcuS_334-543_ | 3.75 | pLeo1243: P_SV40_-DcuS_334-543_-pA | pMS66: P_SV40_-DcuR-VP16-pA | pLeo1228: DcuR-RE_8x_-P_hCMVmin_-SEAP-pA | pDF145 |
|  |  | 50 | 200 | 150 | 350 |
| DcuS_340-543_ | 3.75 | pMS75: P_SV40_-DcuS_340-543_-pA | pMS66: P_SV40_-DcuR-VP16-pA | pLeo1228: DcuR-RE_8x_-P_hCMVmin_-SEAP-pA | pDF145 |
|  |  | 50 | 200 | 150 | 350 |
|  |  |  |  |  |  |
| **Figure 3b** |  |  |  |  |  |
| **label** | **PEI per 6 wells (µg)** | **Plasmid amount (DNA per 6 wells in ng)** | | | |
| **N-terminal acV_H_H fusions** |  | **Receptor** | **Response regulator** | **Reporter** | **Fill up** |
| DcuS_324-543_ | 3.75 | pMS247: P_SV40_-acV_H_H-DcuS_324-543_-pA | pMS66: P_SV40_-DcuR-VP16-pA | pLeo1228: DcuR-RE_8x_-P_hCMVmin_-SEAP-pA | pDF145 |
|  |  | 50 | 200 | 150 | 350 |
| DcuS_330-543_ | 3.75 | pLeo1246: P_SV40_-acV_H_H-DcuS_330-543_-pA | pMS66: P_SV40_-DcuR-VP16-pA | pLeo1228: DcuR-RE_8x_-P_hCMVmin_-SEAP-pA | pDF145 |
|  |  | 50 | 200 | 150 | 350 |
| DcuS_334-543_ | 3.75 | pLeo1247: P_SV40_-acV_H_H-DcuS_334-543_-pA | pMS66: P_SV40_-DcuR-VP16-pA | pLeo1228: DcuR-RE_8x_-P_hCMVmin_-SEAP-pA | pDF145 |
|  |  | 50 | 200 | 150 | 350 |
| DcuS_340-543_ | 3.75 | pLeo1248: P_SV40_-acV_H_H-DcuS_340-543_-pA | pMS66: P_SV40_-DcuR-VP16-pA | pLeo1228: DcuR-RE_8x_-P_hCMVmin_-SEAP-pA | pDF145 |
|  |  | 50 | 200 | 150 | 350 |
| **C-terminal acV_H_H fusions** |  |  |  |  |  |
| DcuS_324-543_ | 3.75 | pLeo1254: P_SV40_-DcuS_324-543_-acV_H_H-pA | pMS66: P_SV40_-DcuR-VP16-pA | pLeo1228: DcuR-RE_8x_-P_hCMVmin_-SEAP-pA | pDF145 |
|  |  | 50 | 200 | 150 | 350 |
| DcuS_330-543_ | 3.75 | pLeo1259: P_SV40_-DcuS_330-543_-acV_H_H-pA | pMS66: P_SV40_-DcuR-VP16-pA | pLeo1228: DcuR-RE_8x_-P_hCMVmin_-SEAP-pA | pDF145 |
|  |  | 50 | 200 | 150 | 350 |
| DcuS_334-543_ | 3.75 | pLeo1260: P_SV40_-DcuS_334-543_-acV_H_H-pA | pMS66: P_SV40_-DcuR-VP16-pA | pLeo1228: DcuR-RE_8x_-P_hCMVmin_-SEAP-pA | pDF145 |
|  |  | 50 | 200 | 150 | 350 |
| DcuS_340-543_ | 3.75 | pLeo1261: P_SV40_-DcuS_340-543_-acV_H_H-pA | pMS66: P_SV40_-DcuR-VP16-pA | pLeo1228: DcuR-RE_8x_-P_hCMVmin_-SEAP-pA | pDF145 |
|  |  | 50 | 200 | 150 | 350 |
| **without ORK** | 3.75 | - | pMS66: P_SV40_-DcuR-VP16-pA | pLeo1228: DcuR-RE_8x_-P_hCMVmin_-SEAP-pA | pDF145 |
|  |  |  | 200 | 150 | 400 |
| **reporter only** | 3.75 | - | - | pLeo1228: DcuR-RE_8x_-P_hCMVmin_-SEAP-pA | pDF145 |
|  |  |  |  | 150 | 650 |
|  |  |  |  |  |  |
| **Figure 4c** |  |  |  |  |  |
| **label** | **PEI per 6 wells (µg)** | **Plasmid amount (DNA per 6 wells in ng)** | | | |
|  |  | **Receptor** | **Response regulator** | **Reporter** | **Fill up** |
| Two-hybrid OGR | 5.4 | pLeo1249: P_CMV_-NLS-acV_H_H-DcuS_324-543_-pA | pAB149: P_hCMV_-DcuR-TetR-pA and pAB150: P_hCMV_-3xNLS-DcuR-VPR-pA | pLeo1017: TetO7_x_-P_hCMVmin_-SEAP-pA | pDF145 |
|  |  | 30 | 120 each | 30 | 600 |
|  |  |  |  |  |  |
| **Figure 5a** |  |  |  |  |  |
| **label** | **PEI per 6 wells (µg)** | **Plasmid amount (DNA per 6 wells in ng)** | | | |
| **N-terminal nanobody fusion** |  | **Receptor** | **Response regulator** | **Reporter** | **Fill up** |
| acV_H_H-DcuS_324-543_ | 5.4 | pMS247: P_SV40_-acV_H_H-DcuS_324-543_-pA | pMS66: P_SV40_-DcuR-VP16-pA | pLeo1228: DcuR-RE_8x_-P_hCMVmin_-SEAP-pA | pDF145 |
|  |  | 50 | 200 | 150 | 350 |
| acV_H_H-DcuS_330-543_ | 5.4 | pLeo1246: P_SV40_-acV_H_H-DcuS_330-543_-pA | pMS66: P_SV40_-DcuR-VP16-pA | pLeo1228: DcuR-RE_8x_-P_hCMVmin_-SEAP-pA | pDF145 |
|  |  | 50 | 200 | 150 | 350 |
| **Figure 5b** |  |  |  |  |  |
| **label** | **PEI per 6 wells (µg)** | **Plasmid amount (DNA per 6 wells in ng)** | | | |
| **C-terminal nanobody fusion** |  | **Receptor** | **Response regulator** | **Reporter** | **Fill up** |
| DcuS_324-543_-acV_H_H | 5.4 | pLeo1254: P_SV40_-DcuS_324-543_-acV_H_H-pA | pMS66: P_SV40_-DcuR-VP16-pA | pLeo1228: DcuR-RE_8x_-P_hCMVmin_-SEAP-pA | pDF145 |
|  |  | 50 | 200 | 150 | 350 |
| acV_H_H-DcuS_330-543_-acV_H_H | 5.4 | pLeo1259: P_SV40_-DcuS_330-543_-acV_H_H-pA | pMS66: P_SV40_-DcuR-VP16-pA | pLeo1228: DcuR-RE_8x_-P_hCMVmin_-SEAP-pA | pDF145 |
|  |  | 50 | 200 | 150 | 350 |
|  |  |  |  |  |  |
| **Figure 5c** |  |  |  |  |  |
| **label** | **PEI per 6 wells (µg)** | **Plasmid amount (DNA per 6 wells in ng)** | | | |
|  |  | **Receptor** | **Response regulator** | **Reporter** | **Fill up** |
| Two-hybrid OGR | 5.4 | pLeo1249: P_CMV_-NLS-acV_H_H-DcuS_324-543_-pA | pAB149: P_hCMV_-DcuR-TetR-pA and pAB150: P_hCMV_-3xNLS-DcuR-VPR-pA | pLeo1017: TetO7_x_-P_hCMVmin_-SEAP-pA | pDF145 |
|  |  | 30 | 120 each | 30 | 600 |
| **Figure 6b** |  |  |  |  |  |
| **label** | **PEI per 6 wells (µg)** | **Plasmid amount (DNA per 6 wells in ng)** | | | |
|  |  | **Receptor** | **Response regulator** | **Reporter** | **Fill up** |
| N-and C-terminal nanobody fusion | 5.4 | pLeo1286: P_SV40_-acV_H_H-DcuS_324-543_-acV_H_H-pA | pMS66: P_SV40_-DcuR-VP16-pA | pLeo1228: DcuR-RE_8x_-P_hCMVmin_-SEAP-pA | pDF145 |
|  |  | 50 | 200 | 150 | 350 |
| **Figure 7b** |  |  |  |  |  |
| **label** | **PEI per 6 wells (µg)** | **Plasmid amount (DNA per 6 wells in ng)** | | | |
|  |  | **Receptor** | **Response regulator** | **Reporter** | **Fill up** |
| EnvZ mutant | 5.4 | pMSx119: P_sv40__EnvZ_232-450;GQEMP:AS_-pA | pMSx64: P_SV40_-OmpR-VP16-pA | pMSx4: OmpR-RE_2x_-P_hCMVmin_-SEAP-pA | - |
|  |  | 300 | 300 | 150 |  |
| EnvZ ORK | 5.4 | pMSx132: P_sv40__acV_H_H-EnvZ_232-450;GQEMP:AS_ -pA | pMSx64: P_SV40_-OmpR-VP16-pA | pMSx4: OmpR-RE_2x_-P_hCMVmin_-SEAP-pA | - |
|  |  | 300 | 300 | 150 |  |
| **Figure 7d** |  |  |  |  |  |
| **label** | **PEI per 6 wells (µg)** | **Plasmid amount (DNA per 6 wells in ng)** | | | |
|  |  | **Receptor** | **Response regulator** | **Reporter** | **Fill up** |
| NarX_263-598_ | 5.4 | PMSx78: P_SV40_-NarX_263-598_-pA | pMSx65: P_SV40_-NarX-VP16-pA | pMSx5: NarX-RE_2x_-P_hCMVmin_-SEAP-pA | - |
|  |  | 300 | 300 | 150 |  |
| NarX_233-598_ | 5.4 | pMSx88: P_SV40_-NarX_233-598_-pA | pMSx65: P_SV40_-NarX-VP16-pA | pMSx5: NarX-RE_2x_-P_hCMVmin_-SEAP-pA | - |
|  |  | 300 | 300 | 150 |  |
| NarX - ORK | 5.4 | PMSx245: P_SV40_-acV_H_H-NarX_263-598_-pA | pMSx65: P_SV40_-NarX-VP16-pA | pMSx5: NarX-RE_2x_-P_hCMVmin_-SEAP-pA | - |
|  |  | 300 | 300 | 150 |  |
|  |  |  |  |  |  |
| **Supplementary Figure 1** |  |  |  |  |  |
| **label** | **PEI per 6 wells (µg)** | **Plasmid amount (DNA per 6 wells in ng)** |  |  |  |
|  |  | **Receptor** | **Response regulator** | **Reporter** | **Fill up** |
| FRB-DcuS ORK  (negative control) | 5.4 | pMSx228: P_SV40_-FRB-DcuS_324-543_-acV_H_H-pA | pMS66: P_SV40_-DcuR-VP16-pA | pLeo1228: DcuR-RE_8x_-P_hCMVmin_-SEAP-pA | pDF145 |
|  |  | 100 | 200 | 150 | 300 |
| FRB-DcuS ORK +  FKBP DcuS ORK | 5.4 | pMSx228: P_SV40_-FRB-DcuS_324-543_-acV_H_H-pA and pMS230: P_SV40_-FKBP-DcuS_324-543_-acV_H_H-pA | pMS66: P_SV40_-DcuR-VP16-pA | pLeo1228: DcuR-RE_8x_-P_hCMVmin_-SEAP-pA | pDF145 |
|  |  | 50 each | 200 | 150 | 300 |
|  |  |  |  |  |  |
| **Supplementary Figure 2** |  |  |  |  |  |
| **label** | **PEI per 6 wells (µg)** | **Plasmid amount (DNA per 6 wells in ng)** |  |  |  |
|  |  | **Receptor** | **Response regulator** | **Reporter** | **Fill up** |
| acV_H_H-DcuR fusion | 5.4 | - | pLeox1237: P_SV40_-acV_H_H-DcuR-VP16-pA | pLeo1228: DcuR-RE_8x_-P_hCMVmin_-SEAP-pA | pDF145 |
|  |  |  | 200 | 150 | 400 |
|  |  |  |  |  |  |
| **Supplementary Figure 3** |  |  |  |  |  |
| **label** | **PEI per 6 wells (µg)** | **Plasmid amount (DNA per 6 wells in ng)** | | | |
|  |  | **Receptor** | **Response regulator** | **Reporter** | **Fill up** |
| Response time | 5.4 | pLeo1286: P_SV40_-acV_H_H-DcuS_324-543_-acV_H_H-pA | pMS66: P_SV40_-DcuR-VP16-pA | pLeo1228: DcuR-RE_8x_-P_hCMVmin_-SEAP-pA | pDF145 |
|  |  | 50 | 200 | 150 | 350 |
|  |  |  |  |  |  |
| **Supplementary Figure 4a** |  |  |  |  |  |
| **label** | **PEI per 6 wells (µg)** | **Plasmid amount (DNA per 6 wells in ng)** | | | |
|  |  | **Receptor** | **Response regulator** | **Reporter** | **Fill up** |
| HeLa | 5.4 | pMS247: P_SV40_-acV_H_H-DcuS_324-543_-pA | pMS66: P_SV40_-DcuR-VP16-pA | pLeo1228: DcuR-RE_8x_-P_hCMVmin_-SEAP-pA | pDF145 |
|  |  | 50 | 200 | 150 | 350 |
|  |  |  |  |  |  |
| **Supplementary Figure 4b** |  |  |  |  |  |
| **label** | **PEI per 6 wells (µg)** | **Plasmid amount (DNA per 6 wells in ng)** | | | |
|  |  | **Receptor** | **Response regulator** | **Reporter** | **Fill up** |
| hMSC-TERT | 5.4 | pMS247: P_SV40_-acV_H_H-DcuS_324-543_-pA | pMS66: P_SV40_-DcuR-VP16-pA | pLeo1228: DcuR-RE_8x_-P_hCMVmin_-SEAP-pA | pDF145 |
|  |  | 50 | 200 | 150 | 350 |
|  |  |  |  |  |  |
| **Supplementary Figure 4c** |  |  |  |  |  |
| **label** | **Lipofectamine Stem transfection reagent** | **Plasmid amount (DNA per 6 wells in ng)** | | | |
|  | **per 6 wells of a 24 well plate (µg)** | **Receptor** | **Response regulator** | **Reporter** | **Fill up** |
| hiPSC | 9 | pMS247: P_SV40_-acV_H_H-DcuS_324-543_-pA | pMS66: P_SV40_-DcuR-VP16-pA | pLeo1228: DcuR-RE_8x_-P_hCMVmin_-SEAP-pA | pDF145 |
|  |  | 200 | 800 | 600 | 1400 |
|  |  |  |  |  |  |
| **Supplementary Figure 4d** |  |  |  |  |  |
| **label** | **PEI per 6 wells (µg)** | **Plasmid amount (DNA per 6 wells in ng)** | | | |
|  |  | **Receptor** | **Response regulator** | **Reporter** | **Fill up** |
| DcuR reporter | 5.4 | pLeo1286: P_SV40_-acV_H_H-DcuS_324-543_-acV_H_H-pA | pMS66: P_SV40_-DcuR-VP16-pA | pLeo1228: DcuR-RE_8x_-P_hCMVmin_-SEAP-pA | pDF145 |
|  |  | 50 | 200 | 150 | 350 |
|  |  |  |  |  |  |
| **Supplementary Figure 4e** |  |  |  |  |  |
| **label** | **PEI per 6 wells (µg)** | **Plasmid amount (DNA per 6 wells in ng)** | | | |
|  |  | **Receptor** | **Response regulator** | **Reporter** | **Fill up** |
| NF-κB reporter | 5.4 | pLeo1286: P_SV40_-acV_H_H-DcuS_324-543_-acV_H_H-pA | pMS66: P_SV40_-DcuR-VP16-pA | pKR32: P_NF-κB_-SEAP-pA | pDF145 |
|  |  | 50 | 200 | 150 | 350 |
|  |  |  |  |  |  |
| **Supplementary Figure 4f** |  |  |  |  |  |
| **label** | **PEI per 6 wells (µg)** | **Plasmid amount (DNA per 6 wells in ng)** | | | |
|  |  | **Receptor** | **Response regulator** | **Reporter** | **Fill up** |
| cAMP reporter | 5.4 | pLeo1286: P_SV40_-acV_H_H-DcuS_324-543_-acV_H_H-pA | pMS66: P_SV40_-DcuR-VP16-pA | pCK53: P_Cre_-SEAP-pA | pDF145 |
|  |  | 50 | 200 | 150 | 350 |
|  |  |  |  |  |  |
| **Supplementary Figure 4g** |  |  |  |  |  |
| **label** | **PEI per 6 wells (µg)** | **Plasmid amount (DNA per 6 wells in ng)** | | | |
|  |  | **Receptor** | **Response regulator** | **Reporter** | **Fill up** |
| NFAT reporter | 5.4 | pLeo1286: P_SV40_-acV_H_H-DcuS_324-543_-acV_H_H-pA | pMS66: P_SV40_-DcuR-VP16-pA | pMX57: P_NFAT_-SEAP-pA | pDF145 |
|  |  | 50 | 200 | 150 | 350 |
|  |  |  |  |  |  |
| **Supplementary Figure 4h** |  |  |  |  |  |
| **label** | **PEI per 6 wells (µg)** | **Plasmid amount (DNA per 6 wells in ng)** | | | |
|  |  | **Receptor** | **Response regulator** | **Reporter** | **Fill up** |
| STAT3 reporter | 5.4 | pLeo1286: P_SV40_-acV_H_H-DcuS_324-543_-acV_H_H-pA | pMS66: P_SV40_-DcuR-VP16-pA | pTS566: P_STAT3_-SEAP-pA | pDF145 |
|  |  | 50 | 200 | 150 | 350 |
|  |  |  |  |  |  |
| **Supplementary Figure 4i** |  |  |  |  |  |
| **label** | **PEI per 6 wells (µg)** | **Plasmid amount (DNA per 6 wells in ng)** | | | |
|  |  | **Receptor** | **Response regulator** | **Reporter** | **Fill up** |
| MAPK reporter | 5.4 | pLeo1286: P_SV40_-acV_H_H-DcuS_324-543_-acV_H_H-pA | pMS66: P_SV40_-DcuR-VP16-pA | PMF111: P_tetO7_-SEAP-pA and  MKP37: P_hCMV_-TetR-Elk1-pA | pDF145 |
|  |  | 50 | 200 | 75 and 100 ng | 325 |
|  |  |  |  |  |  |
| **Supplementary Figure 5** |  |  |  |  |  |
| **label** | **PEI per 6 wells (µg)** | **Plasmid amount (DNA per 6 wells in ng)** | | | |
|  |  | **Receptor** | **Response regulator** | **Reporter** | **Fill up** |
| ORK/OGR/Reporter | 5.4 | pMS247: P_SV40_-acV_H_H-DcuS_324-543_-pA | pMS66: P_SV40_-DcuR-VP16-pA | pLeo1228: DcuR-RE_8x_-P_hCMVmin_-SEAP-pA | pDF145 |
|  |  | 50 | 200 | 150 | 350 |
| OGR/Reporter | 5.4 | - | pMS66: P_SV40_-DcuR-VP16-pA | pLeo1228: DcuR-RE_8x_-P_hCMVmin_-SEAP-pA | pDF145 |
|  |  |  | 200 | 150 | 400 |
| WT control | - | - | - | - | - |
| **Supplementary Figure 6** |  |  |  |  |  |
| **label** | **PEI per 6 wells (µg)** | **Plasmid amount (DNA per 6 wells in ng)** | | | |
|  |  | **Receptor** | **Response regulator** | **Reporter** | **Fill up** |
| EnvZ_232-450_ | 5.4 | pMSx80 | pMSx64: P_SV40_-OmpR-VP16-pA | pMSx4: OmpR-RE_2x_-P_hCMVmin_-SEAP-pA | - |
|  |  | 300 | 300 | 150 |  |

**Supplementary Figures**

**

**Supplementary Figure 1.** *FRB/FKBP POST.* Effect of rapamycin on HEK-293T cells transfected with FRB/FKBP ORKs, OGR and reporter. FRB-ORKs alone were included as the negative control, as this construct does not dimerize in response to rapamycin.

The figure shows the mean ± s.d. of n = 3 biologically independent samples measured 24 h after induction, and is representative of three independent experiments. The bars are each overlaid with a scatter dot plot of the original data points. Source data are provided as a Source Data file.

**

**Supplementary Figure 2.** *Dimerization-dependent DNA binding of DcuR.* Caffeine induced reporter activation of HEK-293T cells expressing acV_H_H-DcuR-VP16 without any ORK.

The figure shows the mean ± s.d. of n = 3 biologically independent samples measured 24 h after induction, and is representative of three independent experiments. The bars are each overlaid with a scatter dot plot of the original data points. Source data are provided as a Source Data file.

**

**Supplementary Figure 3.** *Response time of POST.* Response time of caffeine-induced reporter gene expression for the dual-ORK/DcuR-VP16 system, 2-10 hours after induction.

The figure shows the mean ± s.d. of n = 3 biologically independent samples at the indicated time points, and is representative of three independent experiments. Only error bars larger than the symbol size are displayed. Significance was calculated for SEAP values compared between induced vs. noninduced groups at each time point. The Holm-Sidak method was used. This method consists of multiple two-sided T-tests and corrects for multiple comparisons. *P ≤ 0.05, **P ≤ 0.01, ***P ≤ 0.001, n.s. P > 0.05. Exact P values, t-ratios and degrees of freedom are provided in Supplementary Table S3. Source data are provided as a Source Data file.

**

**Supplementary Figure 4.** *Orthogonality of the POST system.* (**A**) POST performance in Hela, (**B**) hMSC-TERT, and (**C**) iPSCs, compared to HEK-293T cells in panel D. (**D**) Effect of inducers of endogenous pathways in HEK-293T cells on transgene expression from the DcuR reporter plasmid. (**E-I**) Effect of POST activity on endogenous signalling in HEK-293T cells transfected with pathway-specific reporter plasmids and ORK/OGR expression plasmids compared to strong known activators of endogenous signalling pathways to confirm reporter functionality and POST orthogonality. Caffeine was used to activate ORKs.

The panels show the mean ± s.d. of n = 3 biologically independent samples measured 24 h after induction, and are representative of three independent experiments. The bars are overlaid with a scatter dot plot of the original data points. Source data are provided as a Source Data file.

**Supplementary Figure 5.** *Probing for POST toxicity.* Cell proliferation assay for HEK-293T cells transfected with either the full POST system (left), with only OGR and reporter, without ORK (middle) or WT cells without transfection (right). Conversion of WST-8 molecules to an orange formazan dye by metabolically active cells was measured at 450 nm. The measured absorbance is therefore proportional to the number of metabolically active cells.

The figure shows the mean ± s.d. of n = 3 biologically independent samples, and is representative of 2 independent experiments. WST-8 was added 24 h after induction with caffeine and the absorbance at 450 nM was measured 4 h later. The bars are overlaid with a scatter dot plot of the original data points. Source data are provided as a Source Data file.

**Supplementary Figure 6.** *Basal activity of EnvZ_232-450_.* OmpR reporter gene expression of HEK-293T cells transfected with EnvZ_232-450_ and OmpR-VP16. The figure shows the mean ± s.d. of n = 3 biologically independent samples measured 24 h after induction, and is representative of 3 independent experiments. The bar is overlaid with a scatter dot plot of the original data points. Source data are provided as a Source Data file.

Source Data. (separate file)

The Source Data file contains original data for all figures and repeats and is provided as an Excel file.
