# Supplementary material for: Phosphoregulated orthogonal signal transduction in mammalian cells
Source: Nat Commun. 2020 Jun 18;11:3085. doi: 10.1038/s41467-020-16895-1 (PMC7303213; doi:10.1038/s41467-020-16895-1)
Supplement: Supplementary file 2 — Reporting Summary [file 41467_2020_16895_MOESM2_ESM.pdf]

nature research

Corresponding author(s): Martin Fussenegger

Last updated by author(s): May 12, 2020

Reporting Summary

Nature Research wishes to improve the reproducibility of the work that we publish. This form provides structure for consistency and transparency in reporting. For further information on Nature Research policies, see our Editorial Policies and the Editorial Policy Checklist.

Statistics

For all statistical analyses, confirm that the following items are present in the figure legend, table legend, main text, or Methods section.

n/a	Confirmed	
☐	☒	The exact sample size (*n*) for each experimental group/condition, given as a discrete number and unit of measurement
☐	☒	A statement on whether measurements were taken from distinct samples or whether the same sample was measured repeatedly
☐	☒	The statistical test(s) used AND whether they are one- or two-sided<br>*Only common tests should be described solely by name; describe more complex techniques in the Methods section.*
☒	☐	A description of all covariates tested
☐	☒	A description of any assumptions or corrections, such as tests of normality and adjustment for multiple comparisons
☐	☒	A full description of the statistical parameters including central tendency (e.g. means) or other basic estimates (e.g. regression coefficient) AND variation (e.g. standard deviation) or associated estimates of uncertainty (e.g. confidence intervals)
☐	☒	For null hypothesis testing, the test statistic (e.g. *F*, *t*, *r*) with confidence intervals, effect sizes, degrees of freedom and *P* value noted<br>*Give P values as exact values whenever suitable.*
☒	☐	For Bayesian analysis, information on the choice of priors and Markov chain Monte Carlo settings
☒	☐	For hierarchical and complex designs, identification of the appropriate level for tests and full reporting of outcomes
☒	☐	Estimates of effect sizes (e.g. Cohen's *d*, Pearson's *r*), indicating how they were calculated

Our web collection on statistics for biologists contains articles on many of the points above.

Software and code

Policy information about availability of computer code

Data collection	Microsoft Excel for Mac version 16.21
Data analysis	GraphPad Prism version 8.2.1 for MacOS

For manuscripts utilizing custom algorithms or software that are central to the research but not yet described in published literature, software must be made available to editors and reviewers. We strongly encourage code deposition in a community repository (e.g. GitHub). See the Nature Research guidelines for submitting code & software for further information.

Data

Policy information about availability of data

All manuscripts must include a data availability statement. This statement should provide the following information, where applicable:

- Accession codes, unique identifiers, or web links for publicly available datasets
- A list of figures that have associated raw data
- A description of any restrictions on data availability

All data is available in the main text or the supplementary information. Plasmid information is provided in supplementary table 1 and sequence data of original plasmids are deposited at genbank (MT267299-MT267334). Raw data for all figures is provided in a supplementary excel file.

Field-specific reporting

Life sciences study design

All studies must disclose on these points even when the disclosure is negative.

Sample size	No sample size calculation was performed. n = 3 biologically independent samples were predicted to be sufficient for estimating the spread of data, for calculating the mean and for detecting statistically significant differences between compared groups. Preliminary results and experience in similar cell culture experiments support n = 3 biologically independent samples per individual experiment as sufficient to detect meaningful differences in reporter gene expression.
Data exclusions	All data was included.
Replication	All experiments were repeated at least 3 times. All the experimental findings were reproduced and are provided in a supplementary data file.
Randomization	No animal or human research participants were involved. For cell culture experiments no covariates based on sample allocations to experimental groups could be observed and no randomization was performed. All direct comparisons of treated vs. untreated were performed with cells transfected under the same conditions with the same transfection mix and subsequent addition of inducer molecules (e.g. caffeine). The cells for these experiments were cultured in the same plate with the same well-distribution. For most experiments the inner 60 wells of 96 well plates were used and either top 3 or bottom 3 wells were treated with inducers. Inducing the top 3 or bottom 3 wells did not affect gene expression levels.
Blinding	No animal or human research participants were involved. For cell culture experiments investigators were not blinded. Our workflow makes extensive use of multichannel pipetting for conducting several experiments at the same time. Hence it is unlikely that even subconcious bias regarding anticipated results could influence the data. The parallel conduction of several experiments makes it unlikely that the researcher remembers the identity of any given transfection mix while he conducts the experiment, hence elaborate blinding procedures were deemed unnecessary.

Reporting for specific materials, systems and methods

We require information from authors about some types of materials, experimental systems and methods used in many studies. Here, indicate whether each material, system or method listed is relevant to your study. If you are not sure if a list item applies to your research, read the appropriate section before selecting a response.

Materials & experimental systems

n/a	Involved in the study	
☒	☐	Antibodies
☐	☒	Eukaryotic cell lines
☒	☐	Palaeontology and archaeology
☒	☐	Animals and other organisms
☒	☐	Human research participants
☒	☐	Clinical data
☒	☐	Dual use research of concern

Methods

n/a	Involved in the study	
☒	☐	ChIP-seq
☒	☐	Flow cytometry
☒	☐	MRI-based neuroimaging

Eukaryotic cell lines

Policy information about cell lines

Cell line source(s)	HEK-293T (ACC 635) and HeLa (CCL-2) cells were obtained from the German Collection of Microorganisms and Cell Cultures (DSMZ). hiPSCs come from a previous study (doi: 10.1016/j.ymben.2013.02.004) and were derived from the adipose tissue of a 50-year-old patient. They were thawed a few weeks before conducting experiments. hMSC-TERT cells were a gift from Moustapha Kassem (cell line characterization in DOI: 10.1038/nbt0602-592).
Authentication	All cell lines were either recently obtained or thawed from previously verified cryo stocks and no further authentication was performed. hiPSCs were initially characterized by flow cytometry, qRT-PCR, Karyotyping and in vitro differentiation assays (doi: 10.1016/j.ymben.2013.02.004).
Mycoplasma contamination	All cell lines were either recently obtained or thawed and except for hiPSCs they were not further tested for mycoplasma contamination. hiPSCs were tested negative for mycoplasma by IDEXX Bioanalytics, Germany in November 2019.
Commonly misidentified lines (See ICLAC register)	None of the cell lines are listed in the database
